# ABCG2 Overexpression Contributes to Pevonedistat Resistance

**DOI:** 10.3390/cancers12020429

**Published:** 2020-02-12

**Authors:** Rishil J. Kathawala, Claudia M. Espitia, Trace M. Jones, Shariful Islam, Pranav Gupta, Yun-Kai Zhang, Zhe-Sheng Chen, Jennifer S. Carew, Steffan T. Nawrocki

**Affiliations:** 1 Division of Translational and Regenerative Medicine, Department of Medicine, The University of Arizona Cancer Center, Tucson, AZ 85724, USA; rishilkathawala@gmail.com (R.J.K.); espitiac@email.arizona.edu (C.M.E.); tracejones@email.arizona.edu (T.M.J.); shariful@email.arizona.edu (S.I.); jcarew@email.arizona.edu (J.S.C.); 2 Department of Pharmaceutical Sciences, College of Pharmacy and Health Sciences, St. John’s University, Queens, NY 11439, USA; pgupta0@mgh.harvard.edu (P.G.); mail@ykzhang.com (Y.-K.Z.); chenz@stjohns.edu (Z.-S.C.)

**Keywords:** MLN4924, pevonedistat, ABCG2, BCRP, drug resistance

## Abstract

MLN4924 (pevonedistat) is a first-in-class NEDD8-activating enzyme (NAE) inhibitor in clinical trials for the treatment of solid tumors and hematologic malignancies. Despite the promising activity of MLN4924 observed in early trials, drug resistance has been noted in some patients. Identifying the underlying cause of treatment failure may help to better stratify patients that are most likely to benefit from this novel agent. Early preclinical studies revealed that the development of NAEβ mutations promotes resistance to MLN4924. However, these mutations have not been detected in patients that are relapsed/refractory to MLN4924, suggesting that other mechanisms are driving clinical resistance. To better understand the potential mechanisms of MLN4924 resistance, we generated MLN4924-resistant ovarian cancer cells. Interestingly, these cells did not develop mutations in NAEβ. Transcriptome analyses revealed that one of the most upregulated genes in resistant cells was *ABCG2*. This result was validated by quantitative real-time PCR and immunoblotting. Importantly, the sensitivity of MLN4924-resistant cells was restored by lentiviral short hairpin RNA (shRNA) targeting *ABCG2*. Further investigation using ABCG2-overexpressing NCI-H460/MX20 cells determined that these cells are resistant to the anticancer effects of MLN4924 and can be sensitized by co-treatment with the ABCG2 inhibitors YHO-13351 and fumitremorgin C. Finally, HEK293 models with overexpression of wild-type ABCG2 (R482) and variants (R482G and R482T) all demonstrated significant resistance to MLN4924 compared to wild-type cells. Overall, these findings define an important molecular resistance mechanism to MLN4924 and demonstrate that ABCG2 may be a useful clinical biomarker that predicts resistance to MLN4924 treatment.

## 1. Introduction

The small ubiquitin-like molecule NEDD8 plays an important role in controlling the proteasomal degradation of several proteins that are critical for cell survival, oncogenic transformation, and disease progression [1]. Dysregulated NEDDylation has been implicated in malignant pathogenesis and drug resistance, thus providing strong rationale to develop strategies to disrupt this specific mechanism of protein turnover for cancer therapy [2,3,4]. MLN4924 (pevonedistat), a first-in-class inhibitor of NEDD8-activating enzyme (NAE), is a key proximal regulator of NEDDylation that is currently under investigation for the treatment of solid tumors and hematological malignancies [5,6,7]. MLN4924 overcomes multiple mechanisms of pro-survival signaling, triggers DNA damage associated with chromatin licensing and DNA replication factor 1 (CDT1) stabilization and checkpoint kinase 1 (CHK1) activation, and induces stable disease regression [7]. The promising preclinical activity of MLN4924 against multiple cancer types prompted the initiation of early phase clinical trials in patients with leukemia, lymphoma, melanoma, and other malignancies [5,6]. In addition to its anticancer properties, MLN4924 has also been shown to exhibit significant antiviral activity [8,9,10]. Collectively, these results suggest that inhibition of the NEDDylation pathway may have broad therapeutic activity against multiple diseases.

Despite its robust effects in numerous preclinical models and preliminary efficacy in patients with hematologic malignancies, clinical resistance to MLN4924 has been observed [7]. However, the mechanisms that drive MLN4924 resistance remain unclear. Previous studies have demonstrated that treatment-related hetereozygous mutations in the adenosine triphosphate (ATP)-binding pocket and NEDD8-binding cleft of NAEβ promotes acquired resistance to MLN4924 [2,11,12]. Although these mutations effectively contributed to decreased MLN4924 potency in vitro and in vivo, they have not been detected in patients enrolled on clinical trials with MLN4924. It is clear that other currently unknown factors are driving clinical resistance to this agent.

We and other investigators have previously demonstrated that MLN4924 has significant preclinical anticancer activity against ovarian cancer and other malignancies [2,4,7,13,14]. MLN4924 was determined to inhibit cullin NEDDylation, induce DNA re-replication and DNA damage, and induce oxidative stress in ovarian cancer models [4,15,16,17]. These results led to the evaluation of MLN4924 in early clinical trials with patients with advanced solid tumors with limited ovarian cancer patient participation [5,18]. Further investigation of the mechanisms of MLN4924 resistance may yield new strategies, including opportunities for possible patient stratification, to improve overall patient response. To identify potential mechanisms that may confer resistance to MLN4924, we utilized the human ovarian cancer cell line A2780 to create an MLN4924-resistant variant (A2780/MLN-R), analyzed the characteristics of resistant cells, and investigated the mechanisms regulating the resistance to MLN4924. Here, we report the novel finding that the ATP-binding cassette (ABC) transporter ABCG2 contributes to MLN4924 drug resistance in A2780/MLN-R ovarian cancer cells and other cell models where ABCG2 is overexpressed.

## 2. Results

### 2.1. Generation of a Novel MLN4924 Resistant Ovarian Cancer Model System

**Figure 1:**
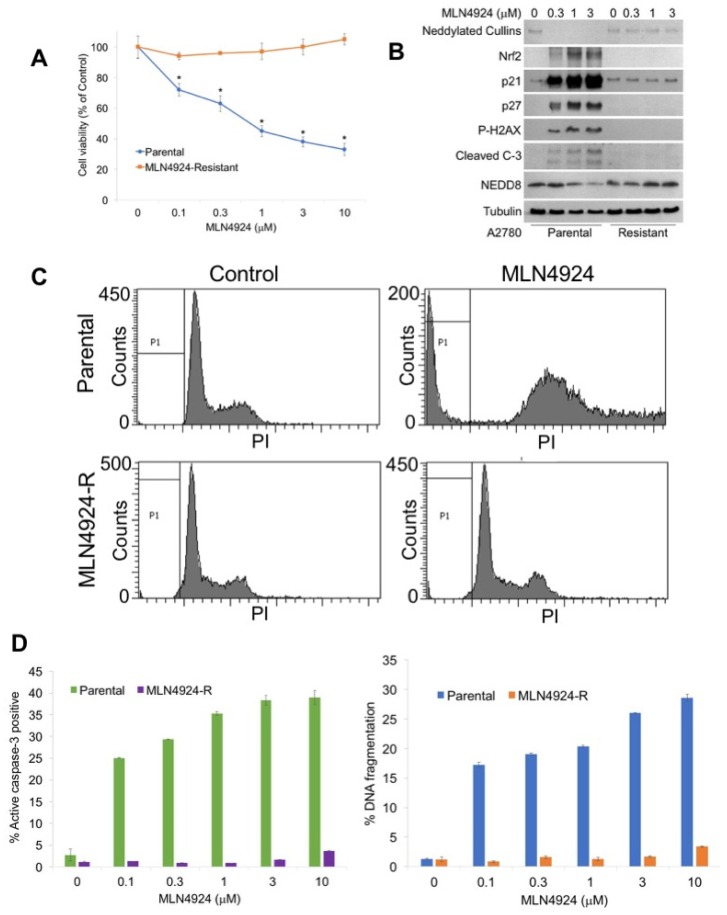
Generation of a novel model of MLN4924 resistance. (**A**) A2780 MLN4924-R cells are significantly less sensitive to MLN4924 treatment than their parental counterparts. Parental and MLN4924-resistant cells were treated with the indicated concentrations of MLN4924 for 72 h. Cell viability was measured by 3-(4,5-dimethylthiazol-2-yl)-2,5-diphenyltetrazolium bromide (MTT) assay. Mean ± SD, *n* = 3. * Indicates a significant difference from resistant cells, *p* < 0.05. (**B**) MLN4924 does not disrupt the NEDDylation cascade in resistant cells. Cells were treated with the indicated concentrations of MLN4924 for 24 h. Protein expression levels were determined by immunoblotting. (**C**) MLN4924 is unable to trigger changes in the cell cycle dynamics in resistant cells. Cells were treated with 10 μM MLN4924 for 48 h. Cell cycle analysis was conducted by PI staining followed by flow cytometry. Representative histograms are shown. (**D**) MLN4924-resistant cells do not undergo apoptosis following MLN4924 treatment. Parental and resistant cells were treated with the indicated concentrations of MLN4924 for 48 h. Apoptosis was determined by PI-FACS analysis (left) and determination of the active caspase-3 levels (right). Mean ± SD, n = 3.

To generate a new model system to study the mechanisms of MLN4924 resistance, A2780 cells were exposed continuously to increasing concentrations of MLN4924 until significant drug resistance (>10 μM) was achieved (A2780/MLN-R) (Figure 1A). The resistant phenotype was stable as cells continued to maintain their resistance in drug-free medium for at least 3 months. The effects of MLN4924 treatment on the levels of selected proteins as well as the disruption of cullin NEDDylation was evaluated by immunoblotting in sensitive and resistant cells. A2780/MLN-R cells were refractory to many of the hallmark features of NAE inhibition, including decreased expression of NEDDylated cullins; induction of p21, p27, and nuclear factor-like 2 (NRF2); and phosphorylation of histone H2AX (Figure 1B). Induction of NRF2 and phosphorylation of H2AX are important markers of oxidative stress and DNA damage, respectively [4,13]. Consistent with the absence of expression of these key factors, cleaved caspase-3 expression was also curtailed in A2780/MLN-R cells (Figure 1B). We next treated parental and resistant cells with MLN4924 to assess its effects on cell cycle distribution by propidium iodide fluorescence activated cell sorting (PI/FACS). Consistent with their drug-resistant phenotype, A2780/MLN-R cells did not accumulate cells in the S phase in the manner that parental and other MLN4924-sensitive cancer cells frequently do (Figure 1C). Accordingly, A2780/MLN-R cells failed to undergo apoptosis following MLN4924 treatment as measured by PI-FACS and active caspase-3 analyses (Figure 1D).

### 2.2. ABCG2 is Highly Upregulated in MLN4924-Resistant Cells

**Figure 2:**
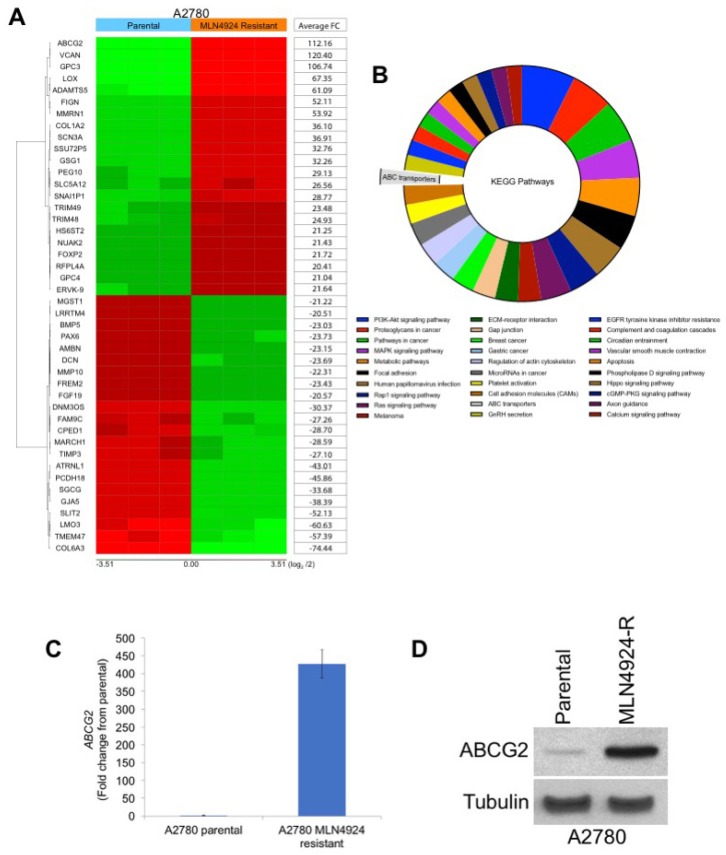
Gene expression analyses identify ABCG2 as a potential factor driving MLN4924 resistance. (**A**) Transcriptome analyses identify *ABCG2* as one of the most upregulated genes in MLN4924-resistant cells. Gene expression changes in parental and resistant A2780 cells were determined using Affymetrix expression arrays. Genes with the most significant induction/repression are illustrated in the heat map. (**B**) Schematic of the significantly altered pathways in MLN4924-resistant cells. The top 30 pathways associated with significantly changed genes by 5-fold or greater (percentage of gene hit against the total number of genes) were analysed using KEGG pathway analysis. (**C**) Quantitative real-time PCR analysis of *ABCG2* levels. *ABCG2* expression in parental and resistant cells was measured by qRT-PCR. Mean ± SD, *n* = 3. (**D**) ABCG2 protein expression is dramatically upregulated in MLN4924-resistant cells. ABCG2 expression was determined in parental and resistant cells by immunoblotting.

As mentioned earlier, various treatment-emergent mutations in NAEβ have been reported to induce resistance to MLN4924 in preclinical models [2,11,12]. To determine whether similar drug-binding site mutations were also driving drug resistance in the A2780/MLN-R cells, we sequenced the NAEβ gene using the methods described by Milhollen et al. [2]. Interestingly, no mutations were detected in the previously reported amino acids 171, 201, 204, 209, and 324 of NAEβ, including the important A171T point mutation. To better understand potential NAEβ-independent mechanisms of MLN4924 resistance, we conducted gene expression profiling on parental and MLN4924-resistant cells. One of the most upregulated genes (112-fold increase) was *ABCG2* (breast cancer resistance protein, BCRP), a well characterized ATP-binding cassette (ABC) transporter that is a key mediator of multidrug resistance (Figure 2A). Analysis of the top pathways significantly changed by 5-fold or greater in MLN4924 resistant cells revealed ABC transporters as significantly upregulated (Figure 2B). The complete gene expression and pathway enrichment analysis is presented in Tables S1–S3. Further analysis of ABCG2 expression by qRT-PCR (Figure 2C) and immunoblotting (Figure 2D) confirmed that ABCG2 was significantly overexpressed in A2780/MLN-R cells.

### 2.3. Targeting ABCG2 Overexpression Diminishes Resistance to MLN4924

**Figure 3:**
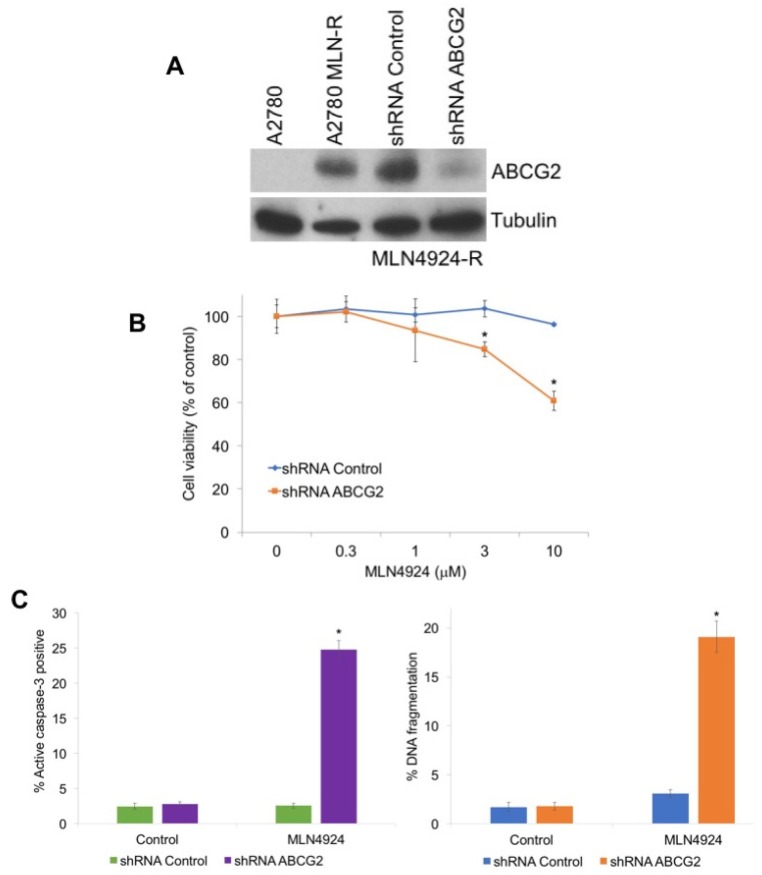
Knockdown of ABCG2 re-sensitizes resistant cells to MLN4924 treatment. (**A**) Knockdown of ABCG2 in MLN4924-resistant cells. A2780/MLN-R cells were infected with non-target control or ABCG2 lentiviral shRNA and positively infected cells were selected with puromycin. Immunoblotting confirmed knockdown of ABCG2 in the resistant cells. (**B**) Knockdown of ABCG2 re-sensitizes resistant cells to MLN4924. A2780/MLN-R cells were infected with control or ABCG2 lentiviral shRNA and were treated with the indicated concentrations of MLN4924 for 72 h. Cell viability was determined by MTT assay. Mean ± SD, n = 3. * Indicates a significant difference compared to non-target control-transfected cells treated with the same concentration. *p* < 0.05. (**C**) Diminished ABCG2 expression sensitizes resistant cells to MLN4924-mediated apoptosis. A2780/MLN-R cells infected with control or ABCG2 lentiviral shRNA were treated with 10 μM MLN4924 for 48 h. Apoptosis was determined by measuring active caspase-3 by flow cytometry and PI-FACS analysis. Mean ± SD, n = 3. * Indicates a significant difference from shRNA control MLN4924-treated cells.

To investigate the role of ABCG2 in MLN4924 resistance, we used shRNA to knockdown its expression in A2780/MLN-R cells, which exhibit high basal ABCG2 levels (Figure 3A). Targeted stable knockdown of ABCG2 rendered A2780/MLN-R cells significantly more sensitive to MLN4924-mediated cell death (Figure 3B,C). Collectively, these results confirm that ABCG2 levels are a key determinant of cellular sensivity to MLN4924.

### 2.4. Mitoxantrone-Selected ABCG2-Overexpressing Cells are Resistant to MLN4924

**Figure 4:**
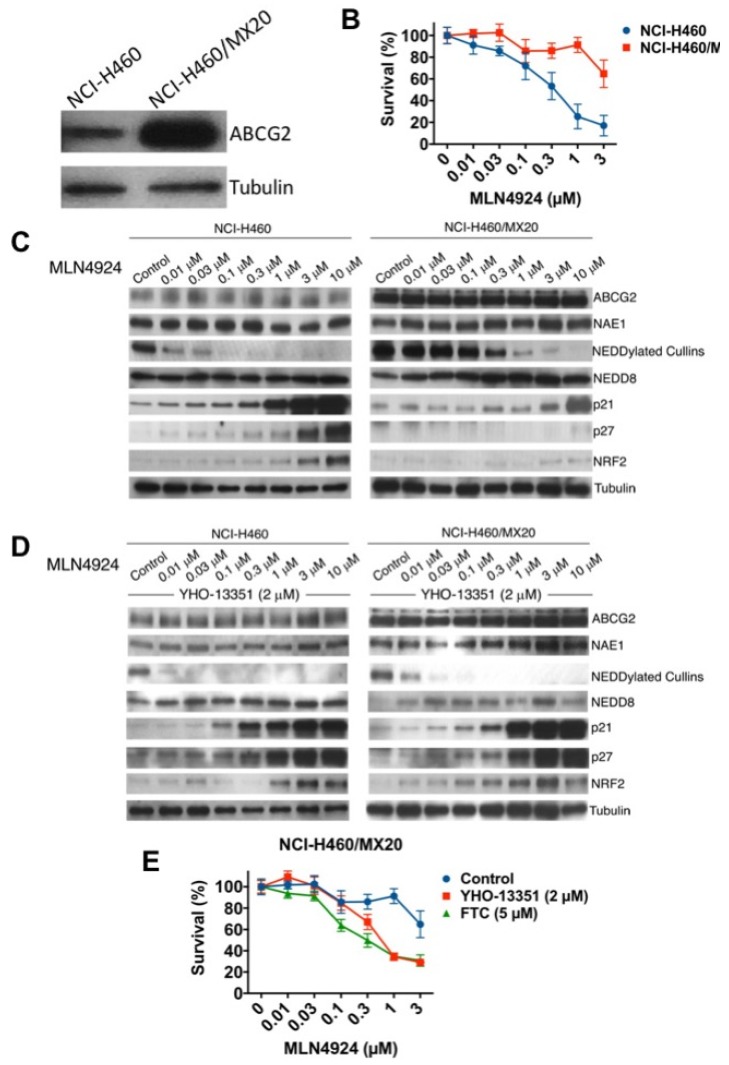
Mitoxantrone-resistant NCI-H460 cells with basal upregulation of ABCG2 are cross-resistant to MLN4924. (**A**) ABCG2 protein expression is upregulated in NCI-H460/MX20 cells compared to NCI-H460 parentals. ABCG2 expression was determined in parental and NCI-H460/MX20 cells by immunoblotting. (**B**) NCI-H460/MX20 cells are resistant to MLN4924 treatment. Parental and resistant cells were treated with the indicated concentrations of MLN4924 for 72 h. Cell viability was measured by MTT assay. Mean ± SD, *n* = 3. (**C**) MLN4924 displays reduced potency in target inhibition and stabilization of downstream NEDD8 substrates, including NRF2, p21, and p27, in NCI-H460/MX20 cells. Cells were treated with the indicated concentrations of MLN4924 for 24 h. Protein expression levels were determined by immunoblotting. (**D**) The ABCG2 inhibitor YHO-13351 overcomes the effects of ABCG2 overexpression. NCI-H460 and NCI-H460/MX20 cells were treated with the indicated concentrations of MLN4924 with or without 2 μM YHO-13351 for 24 h. Protein expression levels were determined by immunoblotting. (**E**) ABCG2 inhibitors YHO-13351 and fumitremorgin C (FTC) enhance the cytotoxic activity of MLN4924 in NCI-H460/MX20 cells. Cells were treated with the indicated concentrations of MLN4924 and 2 μM YHO-13351 or 5 μM FTC for 72 h. Cell viability was determined by MTT assay. Mean ± SD, *n* = 3.

To further establish the mechanistic link between ABCG2 overexpression and resistance to MLN4924, we utilized NCI-H460 non-small cell lung cancer (NSCLC) cells and their mitoxantrone-resistant variants (NCI-H460/MX20) [19]. Consistent with prior findings, basal ABCG2 levels were significantly higher in NCI-H460/MX20 cells compared to their parental counterpart (Figure 4A). In agreement with our ovarian cancer data, the ABCG2-overexpressing NCI-H460/MX20 cells were significantly less sensitive to MLN4924 (Figure 4B). The effects of MLN4924 treatment on the levels of NEDDylated cullins and selected NAE target proteins was also reduced in the NCI-H460/MX20 cells (Figure 4C). Importantly, the diminished effects of MLN4924 on NEDDylated cullins, p21, p27, and NRF2 in resistant cells was reversed by co-treatment with the ABCG2 inhibitor YHO-13351 (Figure 4D). Consistent with these changes in protein expression, YHO-13351 also significantly enhanced the cytotoxicity of MLN4924 against the NCI-H460/MX20 cells (Figure 4E). Similar results were observed following co-treatment of MLN4924 with the related ABCG2 inhibitor fumitremorgin C (FTC) (Figure 4E). Taken together, these results establish that overexpression of ABCG2 may contribute to MLN4924 drug resistance in multiple cancer cell lines.

### 2.5. ABCG2 Overexpression is Sufficient to Confer Resistance to MLN4924

**Figure 5:**
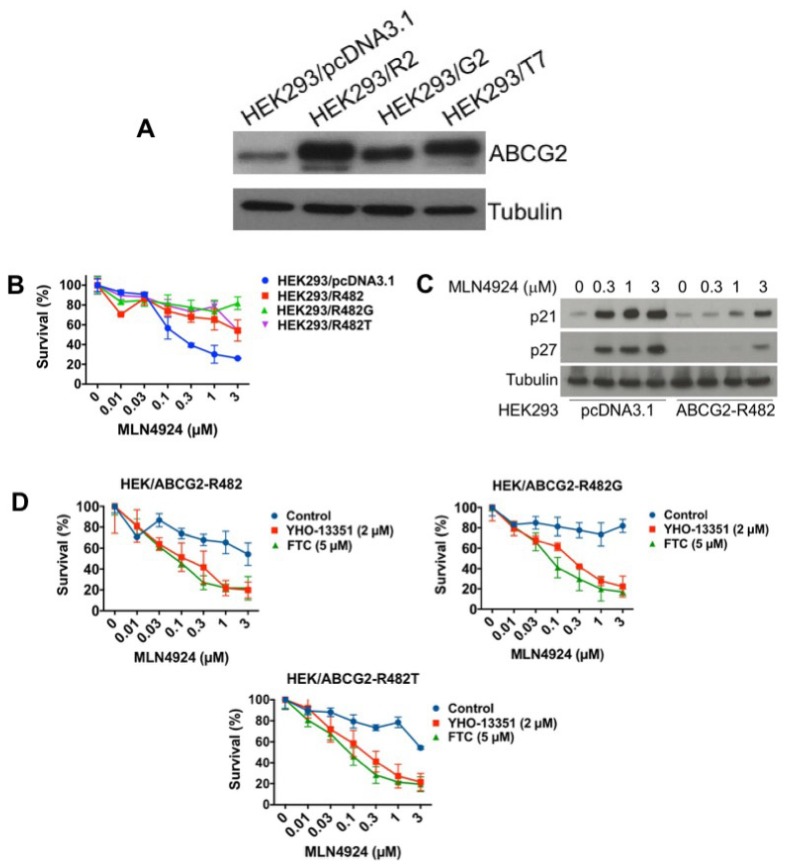
Overexpression of wild-type or mutant ABCG2 confers resistance to MLN4924. (**A**) ABCG2 wild type (R2) and mutants (G2 and T7) were overexpressed in HEK293 cells. ABCG2 expression was determined by immunoblotting. (**B**) ABCG2-overexpressing cells are resistant to MLN4924 treatment. Cells were treated with the indicated concentrations of MLN4924 for 72 h. Cell viability was measured by MTT assay. Mean ± SD, *n* = 3. (**C**) Induction of p21 and p27 is reduced in wild-type ABCG2-overexpressing cells. Cells were treated with the indicated concentrations of MLN4924 for 24 h. Protein expression was determined by immunoblotting. (**D**) The ABCG2 inhibitors YHO-13351 and FTC increase the activity of MLN4924 in ABCG2 wild-type and mutant overexpressing cells. Cells were treated with the indicated concentrations of MLN4924 with or without YHO-13351 and FTC for 72 h. Cell viability was determined by MTT assay. Mean ± SD, *n* = 3.

While our collective data support that overexpression of ABCG2 contributes to MLN4924 resistance in cell lines with acquired drug resistance (A2780/MLN4924 and NCI-H460/mitoxantrone), other mechanisms of drug resistance are likely present in these models that may contribute to its reduced efficacy. To further evaluate the role of ABCG2 in resistance to MLN4924, we investigated the cytotoxicity of MLN4924 in HEK293 cells with overexpression of wild-type *ABCG2* (HEK293/R2) or mutant-*ABCG2* (HEK293/G2 and HEK293/T7) (Figure 5A). While *ABCG2* mutations at position 482 have only been reported in cultured cells (not patient samples), we investigated whether these mutations may alter MLN4924 efficacy. In agreement with our earlier results, overexpression of wild-type or mutant *ABCG2* both significantly decreased the efficacy of MLN4924 (Figure 5B). In addition, upregulation of p21 and p27 was also blunted in the wild-type ABCG2-overexpressing HEK293 cells (Figure 5C). Importantly, co-treatment with ABCG2 inhibitors (YHO-13351 and FTC) reversed resistance to MLN4924 (Figure 5D). This data demonstrates that overexpression of ABCG2 is a key factor that controls sensitivity to MLN4924.

### 2.6. MLN4924 Stimulates ABCG2 ATPase Activity

**Figure 6:**
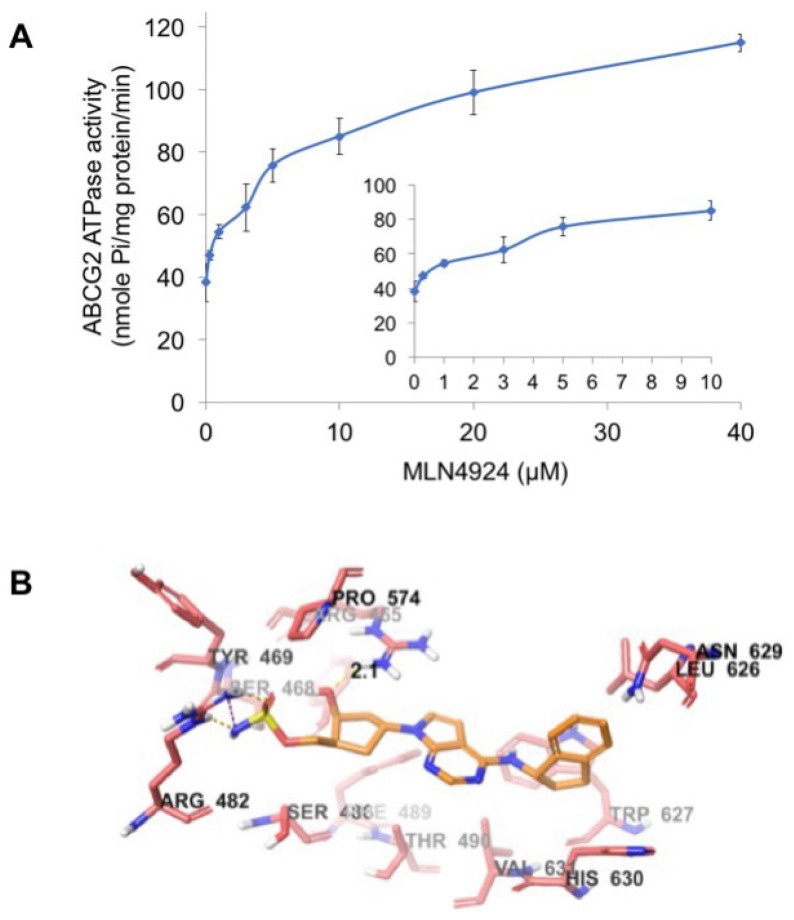
MLN4924 stimulates ABCG2 ATPase activity. (**A**) Effects of various concentrations of MLN4924 on the ATPase activity of ABCG2. The inset graph illustrates the effects of 0–10 μM MLN4924 on the ATPase activity of ABCG2. Mean ± SD, *n* = 3. (**B**) The binding geometry of MLN4924 into the human homology ABCG2-binding pocket. Default CPK coloring was assigned to MLN4924 and nearby residues, expected carbon atoms in MLN4924 were colored orange, and carbon atoms in ABCG2 are colored light red. Dotted yellow lines represent hydrogen bonds and dotted purple lines represent a salt bridge.

ABCG2 utilizes the energy of ATP hydrolysis for the efflux of drug substrates and substrates/modulators either stimulate or inhibit ATPase activity. The stimulatory effect of compounds on the ATPase activity of ABCG2 indicates that the compound is interacting at the drug substrate binding site on the transporter. To investigate potential direct effects of MLN4924 on ABCG2 activity, we measured ABCG2-mediated ATP hydrolysis in the presence of varying concentrations of MLN4924. These analyses showed that there was a 2.58-fold stimulation of ABCG2 basal ATPase activity and the concentration for 50% stimulation was 1.7 µM (Figure 6A). The significant stimulation of ATPase activity observed in this assay suggests that MLN4924 interacts with the ABCG2 drug substrate binding site.

### 2.7. Docking Analysis of MLN4924 with Human ABCG2

To understand the binding interaction of MLN4924 with ABCG2, docking studies were performed. The best scored pose of MLN4924 exhibited a glide score of -7.251 kcal/mol within the transmembrane domain of human homology ABCG2. The hydrophobic scaffold of MLN4924 was stabilized through hydrophobic interactions with nearby residues Tyr464, Ser468, Ser486, Phe489, Thr490, Leu626, Trp627, Asn629, His630, and Val631 (Figure 6B). The 2-hydroxyl group in the cyclopentyl ring formed a hydrogen bond with the side chain of residue Arg465 (HO·· H_2_N-Arg465, 2.1 Å). Moreover, the sulfamic acid group of MLN4924 interacted with ABCG2 via a salt bridge with Arg482 and hydrogen bonding.

## 3. Discussion

MLN4924 has demonstrated promising results in preclinical studies and early clinical trials in both hematological and solid malignancies [2,4,7,13,14,20]. However, the specific factors and/or mechanisms that control clinical sensitivity to MLN4924 remain poorly understood. A key factor in the successful development of any novel investigational drug is discovering which subsets of patients are most likely to respond well to therapy. Beyond this, understanding which disease features positively or negatively affect sensitivity to an individual drug or combination therapy regimen is essential for truly advancing the concept of personalized medicine. The innate heterogeneity of cancers even within a specific histological origin makes this a very challenging endeavor. As soon as MLN4924 was greenlighted for advancement into early phase clinical trials, investigators began efforts to identify specific biomarkers that may regulate response to NAE inhibition. Initial studies attempted to uncover this through the generation of MLN4924-resistant cells in culture systems. Characterization of these resistant lab models revealed the presence of point mutations in the MLN4924 binding site in NAE. Subsequent mechanistic studies confirmed that these mutations were sufficient to confer resitance to MLN4924 [2,11,12]. The next logical step was to evaluate whether these mutations were present in patients that were relapsed/refractory to MLN4924 in clinical trials. Unfortunately, these mutations have not been observed in any patients that relapse following therapy in MLN4924 clinical trials to date. This suggests that other mechanisms are driving clinical resistance to MLN4924.

Here, we created a novel ovarian cancer model of MLN4924 resistance to elucidate mechanisms that diminish its activity. In contrast to other previously generated MLN4924-resistant cancer cell lines, mutations in NAEβ were not detected in our new model. This prompted us to conduct additional analyses to uncover the underlying basis of acquired resistance to MLN4924. Transcriptome analysis revealed a significant elevation of the drug efflux transporter *ABCG2* in MLN4924-resistant cells. This finding, along with its well characterized role in multidrug resistance, led us to hypothesize that ABCG2 overexpression alone may be sufficient to reduce sensitivity to MLN4924. Indeed, knockdown of ABCG2 by shRNA partially restored MLN4924 sensitivity, indicating that it plays a significant role in controlling sensitivity to MLN4924. This was further supported by the reduced efficacy of MLN4924 in multiple ABCG2 overexpression models and the fact that drug sensitivity could be rescued by co-treatment with ABCG2 inhibitors. Although our findings are compelling and worthy of further study, the complex nature of clinical drug resistance makes it essentially inevitable that other mechanisms that are independent of ABCG2 status could be driving the poor response of some patients to MLN4924.

Overexpression of the ABC drug transporter ABCG2 has been linked to acquired resistance to a wide range of clinically relevant anticancer agents and across multiple malignances, including non-small cell lung, thyroid, and breast cancer cells as well as hematological malignancies [21,22,23,24,25]. In addition, overexpression of ABCG2 in esophageal squamous cell carcinoma and advanced non-small cell lung cancer is correlated with significantly decreased overall survival [26,27]. Recent studies also demonstrate that the ABCG2 transporter is upregulated in certain populations of cancer stem cells and normal primitive stem cells in a manner that is correlated with resistance to various antineoplastic drugs [28,29,30,31].

Notably, mutations in *ABCG2* produce distinct substrate preferences within the mutant and wild-type variants. Certain mutations cause conformational changes in the protein and alter the drug binding and efflux capacity of the transporter [32,33]. Mutation of position 482 has the greatest impact on the determination of substrate specificity [34]. The substitution of arginine with threonine or glycine at position 482 produces changes in the substrate profiles among the variants [35]. In our analyses, we showed that overexpression of both the wild-type and mutant forms of ABCG2 confers resistance to MLN4924. Subsequent molecular modeling showed that MLN4924 interacted with the transmembrane domain of ABCG2 and stimulated ABCG2 ATPase activity by acting on the drug substrate binding site.

Our collective findings suggest for the first time that upregulation of ABCG2 is sufficient to significantly diminish the efficacy of MLN4924. Our data support further investigation of ABCG2 as a regulator of sensitivity to this agent, including a rigorous exploration of the multiple potential underlying mechanisms that may culminate in ABCG2 overexpression and the determination of whether high ABCG2 levels are associated with cross-resistance to second generation NAE inhibitors. These results indicate that patients with high ABCG2 levels may not benefit from treatment with MLN4924-based regimens and that it should be considered as a potential exclusion criterion. A high priority should be placed on a statistically powered analysis determining whether basal ABCG2 expression levels are significantly related to clinical outcomes for patients treated on MLN4924 clinical trials. These future studies will enable us to determine and validate whether basal ABCG2 status can be used as a predictive biomarker of clinical response to MLN4924.

## 4. Materials and Methods

### 4.1. Cells and Cell Culture

Human A2780 ovarian cancer cells and the non-small cell lung cancer cell line NCI-H460 were obtained from the American Type Culture Collection (Manassas, VA, USA). A2780, NCI-H460, and its mitoxantrone-selected ABCG2-overexpressing NCI-H460/MX20 variant cells were maintained in RPMI-1640 medium supplemented with 10% fetal bovine serum (FBS) and 1% penicillin/streptomycin in a humidified incubator containing 5% CO_2_ at 37 °C. HEK293/pcDNA3.1, HEK293/R2, HEK293/G2, and HEK293/T7 were established by transfecting HEK293 cells with either empty pcDNA3.1 vector (HEK293/pcDNA3.1), vector containing full-length wild-type ABCG2 (HEK293/R2), or mutant ABCG2 (HEK293/G2 and HEK293/T7) and were cultured in a medium containing 2 mg/mL of G418. Transfected cell lines were grown as adherent monolayers in flasks with Dulbecco’s modified eagle medium (DMEM) supplemented with 10% FBS and 1% penicillin/streptomycin in a humidified incubator containing of 5% CO_2_ at 37 °C. ABCG2-overexpressing and transfected cell lines were kindly provided by Drs. Susan E. Bates and Robert W. Robey (NCI, NIH, Bethesda, MD, USA).

### 4.2. Antibodies and Reagents

Reagents were obtained from: MLN4924 (Cayman Chemical, Ann Arbor, MI), YHO-13351 and fumitremorgin C (FTC), 3-(4,5-dimethylthiazol-2-yl)-2,5-diphenyltetrazolium bromide (MTT), propidium iodide (PI) (Sigma, St. Louis, MO, USA). Antibodies were purchased from the following sources: (anti-NEDD8 [ab81264], anti-γ-H2AX [ab81299], anti-NRF2 [ab137550], and anti-p27 [ab32034] (Abcam, Cambridge, MA, USA), anti-p21 [05-345] (EMD Millipore, Burlington, MA, USA), anti-cleaved caspase-3 [9661] (Cell Signaling Technology, Danvers, MA, USA), anti-β-tubulin [T7816] (Sigma), anti-ABCG2 [sc-58222] (Santa Cruz Biotechnology, Santa Cruz, CA, USA), and sheep anti-mouse-horseradish peroxidase (HRP) and donkey anti-rabbit-HRP (Amersham, Pittsburgh, PA, USA).

### 4.3. Quantification of Drug-Induced Cytotoxicity

Cells were treated with the indicated concentrations of MLN4924 for 72 h. The effects of drug treatment on cell viability were assessed using the MTT assay as previously described [36,37]. Apoptosis was measured by PI staining and fluorescence-activated cell sorting (FACS) analysis of sub-G_0_/G_1_ DNA and quantification of active caspase-3 positive cells by flow cytometry using a commercial kit (BD Biosciences, San Jose, CA, USA).

### 4.4. Mutation Analysis of NAEβ (UBA3) in A2780 and A2780/MLN-R Cells

Total gDNA from A2780 and A2780/MLN-R ovarian cancer cells were isolated using a DNeasy mini kit (Qiagen Inc., Valencia, CA, USA) following the manufacturer’s instructions. DNA was eluted with 100 μL of nuclease-free water. Samples were assessed for concentration and quality with the NanoDrop spectrophotometer. PCR amplifications were conducted using optimized cycling conditions per gene-exon following protocols previously described [2]. Primers used for the reported mutations in positions 171, 201, 204, 209, and 324 are as follows: Exon 8 UBA3 Exon8 Forward primer: TCCCACAAACACACTACCTTCC UBA3 Exon8 Reverse primer: CCTATGAGTCGGTTGTGCTATTG; Exon 9 UBA3 Exon9 Forward primer: CCGACTCATAGGATTACTTGAAAGC UBA3 Exon9 Reverse primer: TCGGTTCATATCTTTCCTCAAATAG; Exon 13 UBA3 Exon13 Forward primer: TGAATCACACAAAACAATGTAAAA UBA3 Exon13 Reverse primer: GAAAATGTATGGGTGACTTTGTTC. After PCR amplification, products from each exon and cell line were resolved on a 1% agarose gel. After confirmation of single bands and correct molecular weight, PCR products were purified using the QIAquick PCR purification kit (Qiagen Inc. Rockville, MD, USA), and prepared for automated sequencing. All samples were sequenced with forward and reverse primers to obtain the complete overlapping sequence of *NAEβ (UBA3)* Exon 8, 9, and 13.

### 4.5. Transcriptome Profiling

A2780 and A2780/MLN-R human ovarian cancer cells were harvested and total RNAs were isolated using the RNeasy Plus Mini Kit (Qiagen Inc.) and treated with the TURBO DNA-free™ Kit (Applied Biosystems, Foster City, CA, USA). In total, 300 ng of total RNA per sample was amplified and hybridized to GeneChip^®^ Human Gene 1.0 ST arrays (Affymetrix, Inc., Santa Clara, CA, USA) according to the manufacturer’s instructions. Affymetrix CEL files were imported into Partek Genomics Suite^®^ 7.18 (Partek Inc., St. Louis, MO, USA) using the default Partek normalization parameters and the robust multi-array average (RMA) analysis adjusted for probe sequence and GC content (GCRMA). Data normalization was performed across all arrays using quantile normalization. Pathway enrichment analysis was performed using the Partek Genomics Suite^®^ software.

### 4.6. Immunoblotting

Cells were incubated with MLN4924 for 24 h as indicated and then lysed for 30 min on ice in Triton X-100 lysis buffer (1% Triton X-100, 150 mmol/L NaCl, 25 mmol/L Tris pH 7.5) with protease inhibitors. Approximately, 50 μg of total cellular protein from each sample were separated by SDS-PAGE. Proteins were transferred to nitrocellulose membranes and blocked with 5% nonfat milk in a tris buffered saline (TBS) solution containing 0.1% Tween-20 for 1 h. The blots were probed overnight with the indicated primary antibodies at 4 °C, washed, and then probed with species-specific secondary antibodies coupled to HRP. Bands were detected by Li-cor chemiluminescence substrate (LI-COR, Lincoln, NE, USA). ImageJ software (NIH, Bethesda, MD, USA) was used for densitometric analysis.

### 4.7. qRT-PCR

cDNA from ovarian cancer cells was used for relative quantification by RT-PCR analyses. First-strand cDNA synthesis was performed from 1 μg of RNA in a 20-μL reaction mixture using the high-capacity cDNA Reverse Transcription Kit (Applied Biosystems, Foster City, CA, USA). *ABCG2* transcripts were amplified using commercially available TaqMan Gene expression assays (Applied Biosystems). *GAPDH* was used as a housekeeping gene.

### 4.8. shRNA Knockdown of ABCG2

A2780/MLN-R human ovarian cancer cells were infected with lentiviral particles containing nontargeted (control) or targeted short hairpin RNA (shRNA) directed at ABCG2 (Santa Cruz Biotechnology) according to the manufacturer’s instructions. Positively infected cells were selected with puromycin treatment. Drug-induced cytotoxicity was quantified by MTT cell viability assay as described previously [38]. Knockdown efficiency was assessed by immunoblotting.

### 4.9. ATPase Activity of ABCG2

The vanadate-sensitive ATPase activity of ABCG2 was determined in membranes treated with MLN4924 at the concentrations of 0 to 40 µM using the PREDEASY ATPase Kits as previously described [39,40].

### 4.10. Molecular Modeling of Human ABCG2 and Docking of MLN4924

Docking simulations of MLN4924 were performed by our previous protocol with slight modifications [41]. Generally, a grid of human homology ABCG2 on the centroid of Arg482 was used for docking on ABCG2. The energy minimized structure of MLN4924 was built in Maestro and prepared by Ligprep 3.4 (Schrödinger, USA, 2015). The energy minimized structure of MLN4924 was subjected to Glide v6.5 XP (extra precision) docking. The top-scoring docked complex of ABCG2-MLN4924 was used for interactions analysis and graphic representation. All computations were carried out on a 6-core Intel Xeon Processor with a Mac OS.

### 4.11. Statistical Analysis

Differences of the parameters between the two cell groups were analyzed by two tailed Student’s unpaired t-test. The a priori significance level was set at *p* < 0.05.

## 5. Conclusions

Here, we demonstrated that ABCG2 overexpression significantly contributes to MLN4924 resistance. Our findings identify a new molecular resistance mechanism to MLN4924 and suggest that ABCG2 expression may be a key clinical biomarker that predicts resistance to MLN4924 treatment.

## Supplementary Materials

The following are available online at https://www.mdpi.com/2072-6694/12/2/429/s1, Table S1: Gene expression changes in A2780 MLN4924-resistant cells vs. parental cells, Table S2: Enriched pathways in A2780 MLN4924-resistant cells vs. parental cells, Table S3: Pathway-enrichment analysis in A2780 MLN4924-resistant cells vs. parental cells. Table S4: Immunoblot densitometry analyses.
